# High-resolution multimodal imaging reveals spatial and temporal heterogeneity of airway mucus plugging in mice with muco-obstructive lung disease

**DOI:** 10.1038/s41598-025-22537-7

**Published:** 2025-11-24

**Authors:** Claudia V. Benke, Julia Duerr, Annika Engel, Christian Dullin, Wolfram Stiller, Heinz Horstmann, Claudia Redenbach, Maximilian Ackermann, Hans-Ulrich Kauczor, Thomas Kuner, Mark O. Wielpütz, Marcus A. Mall, Willi L. Wagner

**Affiliations:** 1 Department of Diagnostic and Interventional Radiology (DIR), Heidelberg University Hospital, Heidelberg, Germany; 2 Translational Lung Research Center (TLRC), German Center for Lung Research (DZL), University Heidelberg, Heidelberg, Germany; 3 Department of Pediatric Respiratory Medicine, Immunology and Critical Care Medicine, Charité - Universitätsmedizin Berlin, Corporate Member of Freie Universität Berlin and Humboldt-Universität, Berlin, Germany; 4 German Center for Lung Research (DZL), Associated Partner Site Berlin, Berlin, Germany; 5 German Center for Child and Adolescent Health (DZKJ), Partner Site Berlin, Berlin, Germany; 6 Department of Mathematics, Technical University of Kaiserslautern (now part of RPTU University Kaiserslautern-Landau), Kaiserslautern, Germany; 7 Hamburg University, Hamburg, Germany; 8 Department of Diagnostic and Interventional Radiology, University Medical Center Goettingen, Goettingen, Germany; 9 Translational Molecular Imaging, Max-Plank-Institute for Multidisciplinary Sciences, Goettingen, Germany; 10 Department of Functional Neuroanatomy, Institute of Anatomy and Cell Biology, Heidelberg University, Heidelberg, Germany; 11 Department of Mathematics, RPTU University Kaiserslautern-Landau, Kaiserslautern, Germany; 12 Institute of Pathology, University Clinics of RWTH University, Aachen, Germany; 13 Institute of Pathology and Molecular Pathology, Helios University Clinic Wuppertal, University of Witten/Herdecke, Witten, Germany; 14 Institute of Anatomy, University Medical Center of the Johannes Gutenberg-University, Mainz, Germany; 15 Diagnostic Radiology and Neuroradiology, University Medicine Greifswald, Greifswald, Germany

**Keywords:** ENaC, Mucus plugging, Cystic fibrosis, Micro-CT, Synchrotron tomography, Airway obstruction, Diseases, Medical research

## Abstract

Mucus plugging is a hallmark of muco-obstructive lung diseases; however, the spatiotemporal dynamics of plug formation remain poorly understood. We used a multimodal high-resolution imaging approach to study mucus plugging in newborn (0–1 days), juvenile (13–16 days), and adult (49–59 days) βENaC-transgenic mice (βENaC-tg, *n* = 24), a model of muco-obstructive lung disease, in comparison to age-matched wild-type mice (*n* = 28). Micro-computed tomography (µCT) enabled whole lung mucus scoring on a scale from 0 (no obstruction) to 2 (> 50% obstruction) using an extended murine airway tree nomenclature. A subset of βENaC-tg lungs (5 juvenile, 5 adult) underwent localised synchrotron radiation-based computed tomography (SRCT) for segmentation-based calculation of mucus area and contact ratios as indicators of luminal obstruction and airway wall adherence. µCT-guided scanning electron microscopy (SEM) provided nanoscale visualisation of mucus plugs on selected βENaC-tg lung samples (3 juvenile). µCT revealed age-dependent, heterogeneous mucus plug formation, with significantly increased mucus scores in juvenile (0.45 ± 0.26) and adult (0.30 ± 0.17) βENaC-tg compared to wild-type mice (0.01 ± 0.02 with *P <* 0.01, and 0.01 ± 0.02 with *P* < 0.05, respectively). SRCT analysis showed a strong positive correlation between mucus area and contact ratios (*r* ≥ 0.80), while SEM revealed the mucus plug ultrastructure. This multimodal imaging approach highlights the spatiotemporal heterogeneity of mucus plugging, forming a basis for future research on targeted therapeutic strategies for muco-obstructive lung diseases.

**Supplementary Information:**

The online version contains supplementary material available at 10.1038/s41598-025-22537-7.

## Introduction

Airway mucus plays a crucial role in lung defence by trapping inhaled particles and pathogens and facilitating mucociliary clearance. In muco-obstructive lung diseases, such as cystic fibrosis (CF), impaired mucociliary clearance, due to increased concentration and cross-linking of mucins, leads to the accumulation of mucus in the airways and the formation of intraluminal mucus plugs^1–5^. Previous studies have shown that excess airway mucus not only causes airflow obstruction and loss of lung function, but is also an important driver of chronic infection and inflammation that perpetuate progressive lung damage^3,6–10^.

To investigate the role of airway mucus obstruction in the pathogenesis of muco-obstructive lung diseases, we previously developed the βENaC-transgenic (βENaC-tg) mouse model, which is characterised by lung-specific overexpression of the β-subunit of the epithelial sodium channel (βENaC), resulting in increased airway sodium and water absorption, depletion of airway surface liquid, increased mucus concentration, reduced mucociliary clearance, and formation of airway mucus plugs, which in turn trigger chronic inflammation and structural lung damage^3,6,7,11–16^. These features closely mimic the pathophysiology observed in patients with CF and other muco-obstructive lung diseases, making the βENaC-tg mouse a tool for studying muco-obstructive lung diseases^1,2,12,17^. Previous studies used light microscopy with standard histological staining techniques to characterise airway narrowing and mucus obstruction in βENaC-tg mice^11,18–21^. These studies revealed age-dependent mucus accumulation, with early postnatal tracheal obstruction, persistent central airway mucus obstruction by three days of age, and progressive involvement of both large and small airways in older mice^11,18–21^. However, traditional histological approaches are limited by their two-dimensional (2D) nature and the risk of sampling bias, particularly given the heterogeneous distribution of mucus plugs in this model^1,19,22–24^. To overcome these limitations, earlier studies used imaging techniques such as flat-panel detector-based volumetric computed tomography (VCT) and micro-computed tomography (µCT) to visualise mucus plugging in three dimensions (3D)^19,22^ and to perform image-based lung function testing^25^. While these methods have shown promise in detecting mucus-related opacities, a comprehensive quantitative analysis of mucus obstruction and distribution throughout the airway tree could not be performed^19,22^.

The aim of the present study was to assess the spatial and age-dependent temporal heterogeneity of airway mucus plug formation in βENaC-tg mice from birth to adult stages using a multimodal, high-resolution experimental imaging approach. Three techniques were employed in a complementary fashion, together with a modified vascular perfusion lung fixation technique to preserve airway mucus in its orthotopic position within the airways, ensuring accurate assessment of mucus distribution and properties across all imaging modalities. This allows for correlative imaging approaches, combining (i) whole organ µCT to assess airway mucus obstruction airway segment-specific, (ii) localised synchrotron radiation-based computed tomography (SRCT) to regionally analyse the location of mucus plugs at the airway cross-sectional level, and (iii) scanning electron microscopy (SEM) to study the ultrastructural characteristics of mucus plugs in the fixed specimen.

We used these techniques to comprehensively map the spatiotemporal characteristics of airway mucus plugs, by integrating imaging modalities that span from whole organ macroscale to ultrastructural nanoscale resolution.

## Results

### Multiscale imaging of airway mucus plugs using µCT and SRCT

To assess airway mucus obstruction across scales, we used a multimodal high-resolution imaging approach in βENaC-tg (*n* = 24) and wild-type (*n* = 28) mice, grouped into newborn (0–1 days), juvenile (13–16 days), and adult (49–59 days) ages (see Table 1). In this study, µCT (9 μm voxel size) was first performed to assess mucus obstruction at the whole organ level. Subsequently, SRCT (3.25 μm voxel size) was performed on a subset of 10 βENaC-tg lungs (5 juvenile, 5 adult), focusing on localised regions of the left lung for higher resolution imaging.

**Table 1:** Characteristics of the study group. Subjects were divided into three groups according to their age: newborn (0–1 days), juvenile (13–16 days), and adult (49–59 days). Age and weight data are presented as mean ± standard deviation. wt = wild-type. βENaC-tg = βENaC-transgenic.

	wt			βENaC-tg		
Age group	Newborn	Juvenile	Adult	Newborn	Juvenile	Adult
Number of mice	11	9	8	7	10	7
Age [days]	0.5 ± 0.5	13.4 ± 0.5	56.3 ± 4.5	0.3 ± 0.5	13.8 ± 0.9	55.7 ± 4.6
Female/male/unknown	4/2/5	3/6/0	5/2/1	2/3/2	4/3/3	4/3/0
Weight [g]	1.4 ± 0.2	7.3 ± 0.5	23.4 ± 3.2	1.4 ± 0.2	6.7 ± 0.7	22.4 ± 2.6

**Fig. 1:**
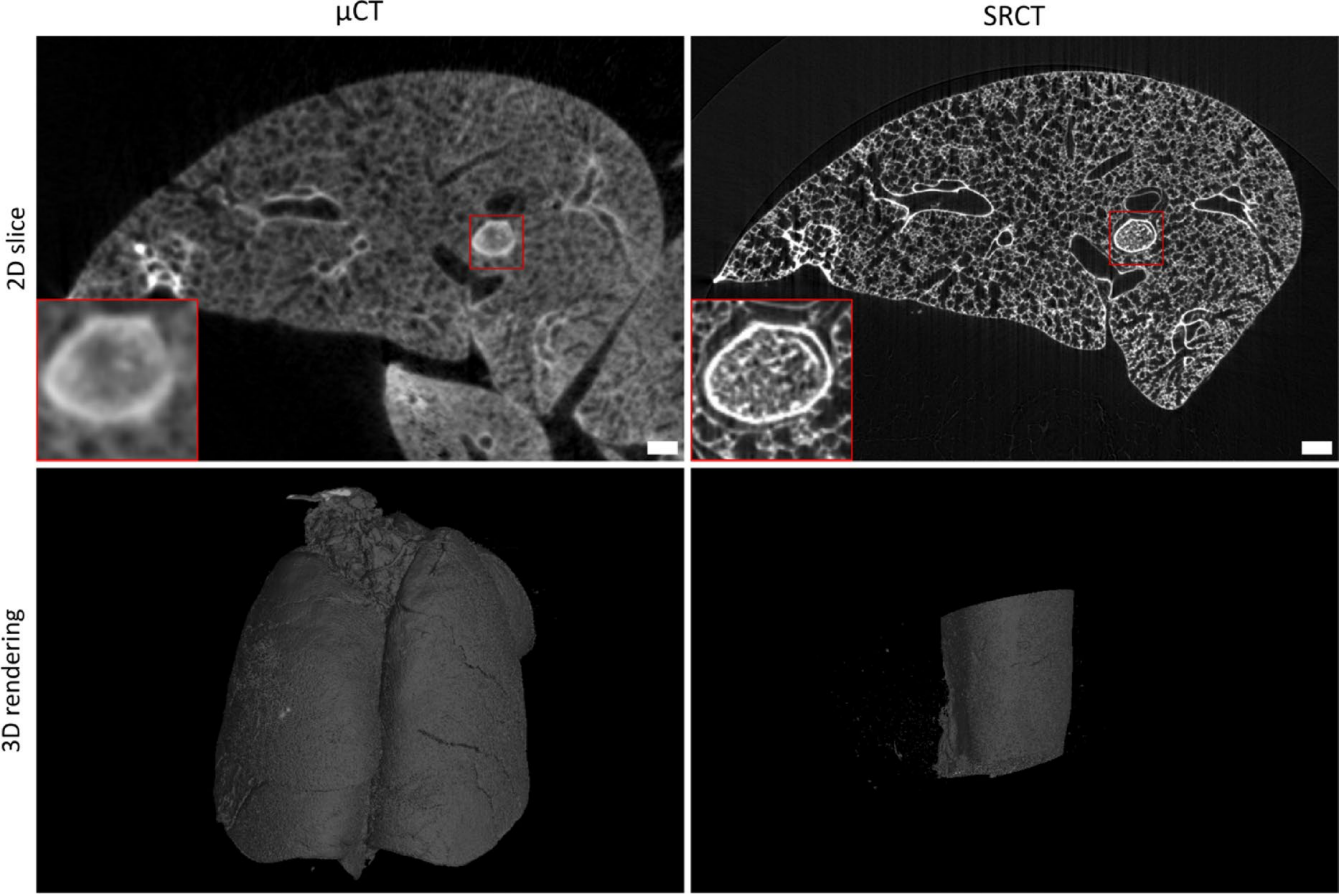
Multimodal high-resolution imaging bridges scales in airway mucus obstruction. Comparative visualisation of airway mucus plugs in βENaC-tg mice using two high-resolution imaging modalities: µCT (9 μm voxel size; left) and SRCT (3.25 μm voxel size; right). The top row displays representative 2D slices from the same βENaC-tg mouse, highlighting the increased level of detail in mucus plug visualisation at higher spatial resolution (regions magnified in red boxes). The bottom row shows 3D volume renderings of the corresponding data sets: the µCT dataset capturing the entire lung and the SRCT dataset capturing a localised region of the left lung due to its smaller field of view. Red boxes indicate areas magnified 3x. Scale bars are 250 μm. βENaC-tg = βENaC-transgenic. µCT = micro-computed tomography. SRCT = synchrotron radiation-based computed tomography. 2D = two-dimensional. 3D = three-dimensional.

Figure 1 shows representative 2D slices (top row) and 3D renderings (bottom row) from both modalities, µCT (left) and SRCT (right), highlighting the difference in field of view between µCT (whole lung) and SRCT scans (localised regions). The Supplementary Video 1 (see data availability) provides a 360° view of these two 3D renderings, and additionally a dynamic cross-sectional visualisation of the µCT and SRCT data sets of the same βENaC-tg mouse, highlighting the increased level of detail in mucus obstruction at higher resolution.

### Age-dependent progression of airway mucus obstruction in βENaC-tg mice

**Fig. 2:**
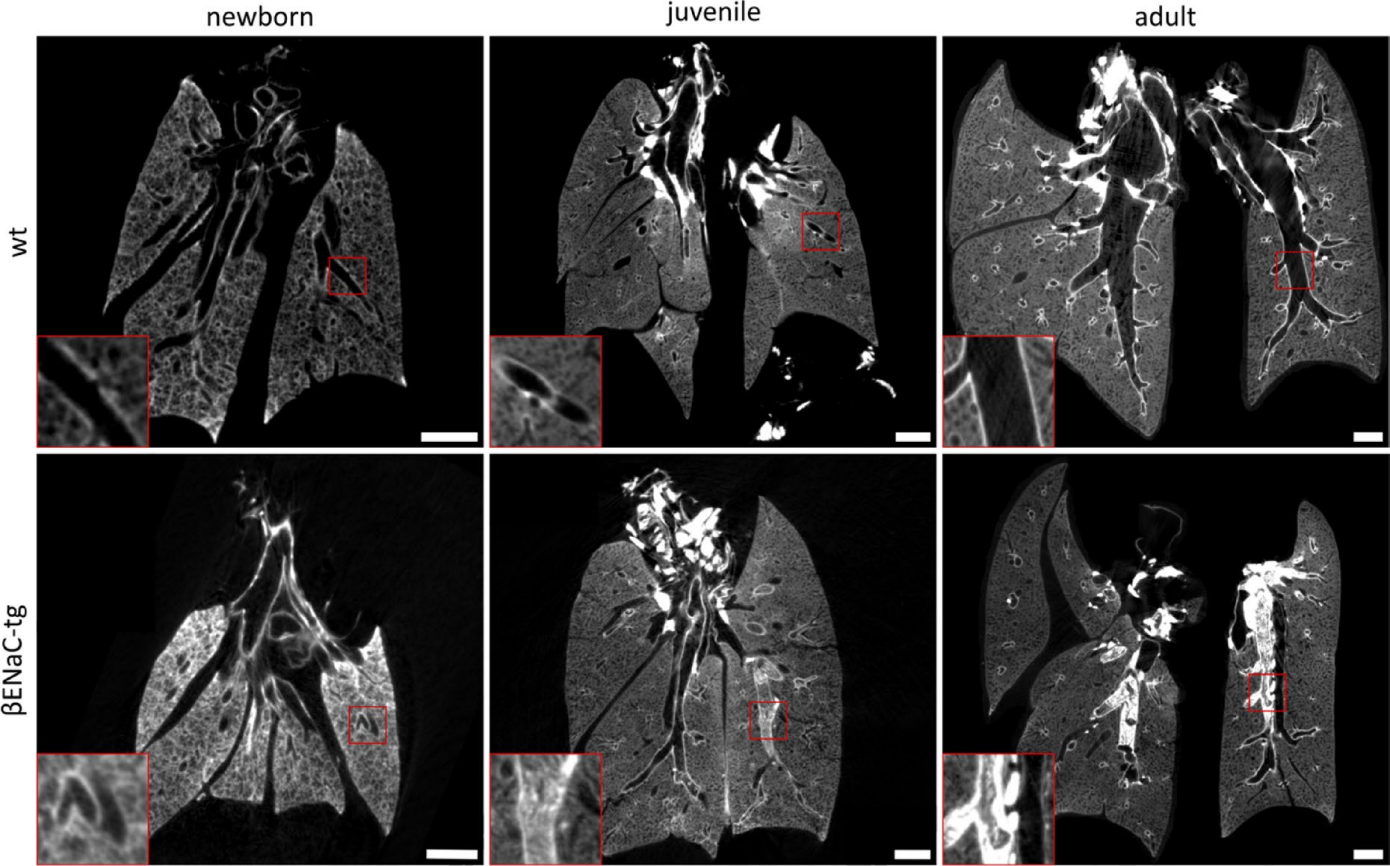
µCT imaging revealed age-dependent progression of airway mucus obstruction in βENaC-tg mice. Representative µCT reconstructions illustrating the age-dependent progression of airway mucus obstruction in βENaC-tg mice, compared to wild-type. Images show newborn, juvenile, and adult stages for both genotypes. Note the spontaneous formation and accumulation of mucus plugs in βENaC-tg mice from juvenile to adult stages, which is absent in wild-type mice. Red boxes mark regions magnified 3x. Scale bars are 1 mm. µCT = micro-computed tomography. wt = wild-type. βENaC-tg = βENaC-transgenic.

**Fig. 3:**
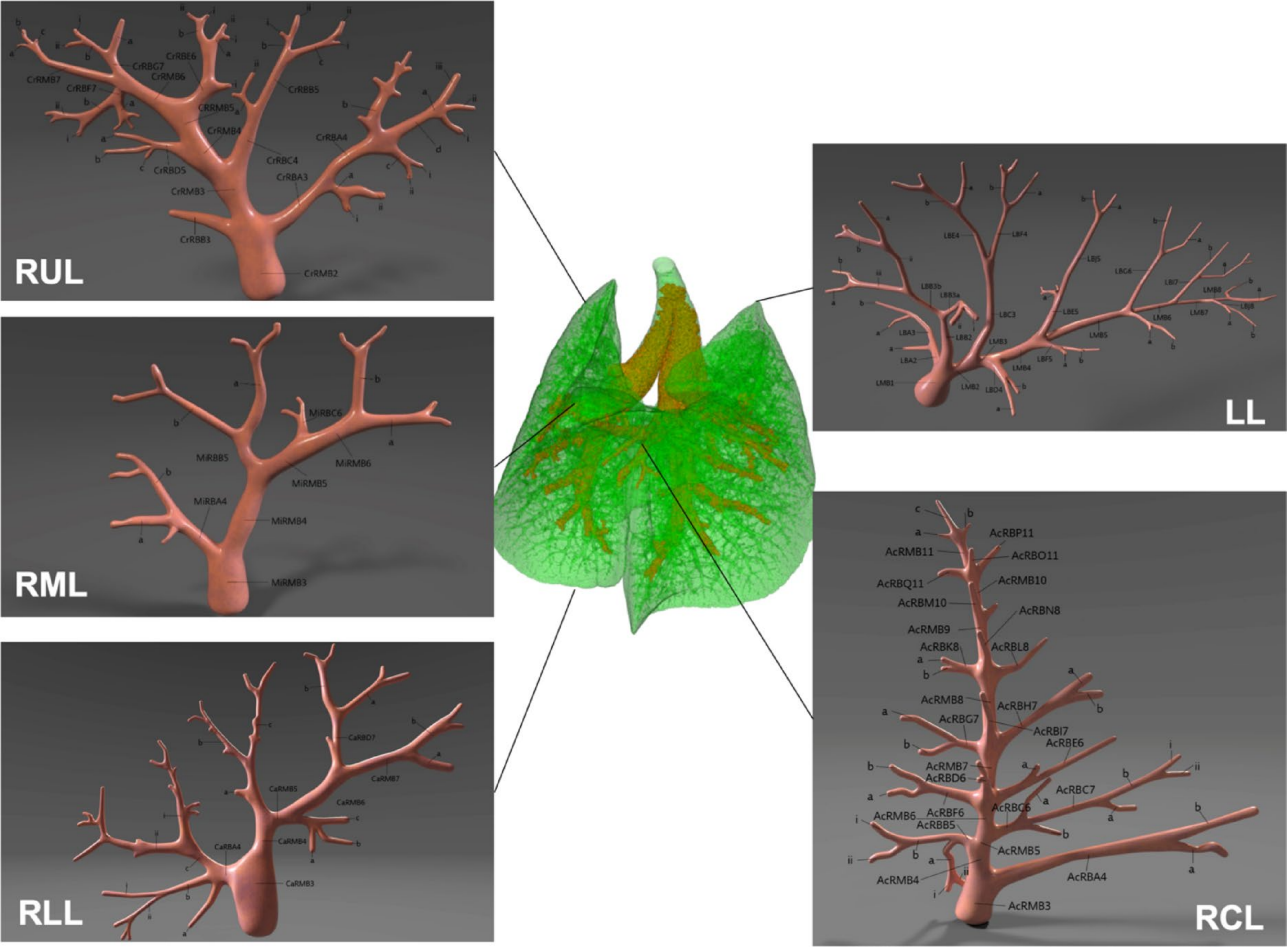
Comprehensive murine airway tree nomenclature for systematic analysis of mucus obstruction. A detailed nomenclature was used for visual mucus scoring, showing the complex branching structure of the murine respiratory system. Airway segments are systematically labelled across all five lung lobes (four right lobes and one left lung) and positioned around a volume-rendered murine lung model (green). Each airway segment is identified by a black font label that specifies lobe, laterality, airway type, and generation number relative to the trachea. Successive branching levels are denoted by capital letters, lowercase letters, and Roman numerals. The nomenclature system is described in detail by Thiesse et al.^26^. RUL = right upper lobe. RML = right middle lobe. RLL = right lower lobe. RCL = right post-caval lobe. LL = left lung.

**Fig. 4:**
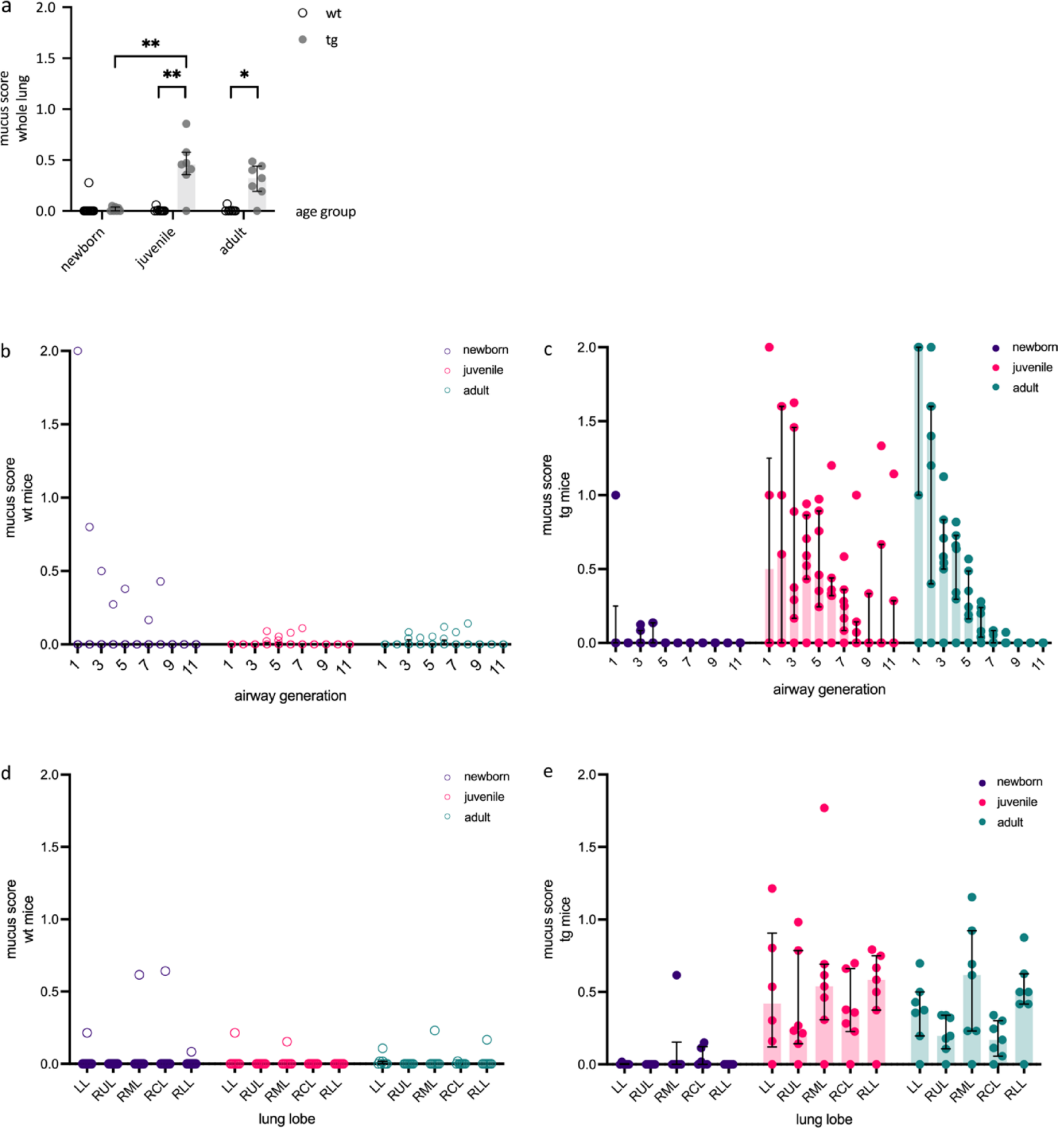
Spatial and temporal characteristics of airway mucus plugs in βENaC-tg mice. Comprehensive analysis of age-dependent airway mucus obstruction from newborn to adult stages reveals heterogeneous nature of mucus accumulation in βENaC-tg mice. Visual mucus scoring was performed on airway segments using micro-computed tomography image datasets, providing insight into the severity and location of mucus plugging for the whole lung (a), the eleven airway generations (b, c), and five lung lobes (d, e), shown for newborn, juvenile, and adult mice respectively. This detailed breakdown allows the identification of age- and region-specific patterns of mucus obstruction in the βENaC-tg mouse model. The inclusion of wild-type controls provided a comparison to highlight the pathological mucus accumulation in the transgenic mice. Data are presented as mean mucus scores, shown as median with interquartile range. LL = left lung. RUL = right upper lobe. RML = right middle lobe. RCL = right post-caval lobe. RLL = right lower lobe. wt = wild-type. tg = βENaC-transgenic. *n* = 6–11 mice/group. *P < 0.05. **P < 0.01.

µCT analysis revealed distinct patterns of airway mucus obstruction in newborn, juvenile, and adult βENaC-tg mice across the whole lung (Fig. 2). Visual scoring was performed by semi-quantitative assessment of airway mucus obstruction at the airway segment level, on a scale of 0 to 2 (0 = no mucus, 1 = mucus obstructing up to 50% of the airway lumen, 2 = mucus obstructing more than 50% of the airway lumen), using an extended mouse airway tree nomenclature (Fig. 3). The nomenclature adopts the systematic labelling of Thiesse et al.^26^, allowing consistent identification of airway segments across samples. It covers airway generations 1–11, allowing detailed analysis of mucus obstruction patterns from proximal to distal airways. The trachea (generation 0) is excluded from visual mucus scoring to focus on the intrapulmonary airways, from carina onward. This comprehensive nomenclature allows accurate segment-level assessment of mucus obstruction throughout the bronchial tree, providing a basis for quantitative analysis of mucus distribution patterns in different lung regions and airway generations. Such detailed mapping is critical for understanding the spatio-temporal dynamics of mucus obstruction in the βENaC-tg mouse model. Figure 4a shows that wild-type mice were characterised by minimal to no mucus obstruction across all age groups, with whole organ mean mucus scores close to zero (newborn: 0.03 ± 0.08, juvenile: 0.01 ± 0.02, adult: 0.01 ± 0.02). In contrast, βENaC-tg mice showed an age-dependent progression of mucus obstruction. Newborn βENaC-tg mice showed whole organ mean mucus scores close to zero (0.02 ± 0.02), comparable to wild-type controls. Juvenile βENaC-tg mice had significantly higher mean mucus scores (0.45 ± 0.26, *P* < 0.01), indicating a significant increase in mucus obstruction and adult βENaC-tg mice maintained elevated mean mucus scores (0.30 ± 0.17, *P* < 0.05), indicating persistent airway mucus accumulation, compared to their respective age-matched wild-type controls.

### Mucus obstruction shows different regional patterns

Analysis of individual lung lobes and airway generations revealed heterogeneous patterns of mucus obstruction (Fig. 4b-e, Supplementary Fig. S1 and S2 online). Wild-type mice showed in general no mucus apposition across all lung lobes and all age groups, with only few outliers (Fig. 4d and Supplementary Fig. S1 online), like newborn βENaC-tg mice (Fig. 4e and Supplementary Fig. S1 online). Juvenile and adult βENaC-tg mice showed significantly increased mean mucus scores across all lung lobes compared to their respective wild-type controls (*P* < 0.05; Fig. 4d-e and Supplementary Fig. S1 online). The degree of airway mucus obstruction was significantly increased in juvenile βENaC-tg mice compared to newborn βENaC-tg mice in all lobes, except for the right middle lobe (*P* < 0.05; Supplementary Fig. S1 online).

Furthermore, wild-type mice showed in general no elevated mean mucus scores for the different airway generations across all age groups, with only few outliers, which showed mucus apposition in generation 1–8 (Fig. 4b and Supplementary Fig. S2online). Few newborn βENaC-tg mice showed some obstruction in proximal airway generations 1–4, however, with no significant difference compared to their wild-type control mice (Fig. 4c and Supplementary Fig. S2 online). Juvenile βENaC-tg mice showed the most extensive and evenly distributed mucus obstruction across all generations, where few mice rated highest scores in proximal generations 1–3, and overall elevated scores were seen in generations 3–7 compared to both their respective wild-type controls and newborn βENaC-tg mice (*P* < 0.01 and *P* < 0.05, respectively; Fig. 4c and Supplementary Fig. S2 online). Adult βENaC-tg mice showed an almost exponential decrease in mucus scores from generation 1 to 8, with significantly higher scores in generations 1–6 compared to their respective wild-type control mice (*P* < 0.05, Fig. 4c and Supplementary Fig. S2 online).

### Correlation between mucus airway wall adherence and luminal obstruction

**Fig. 5:**
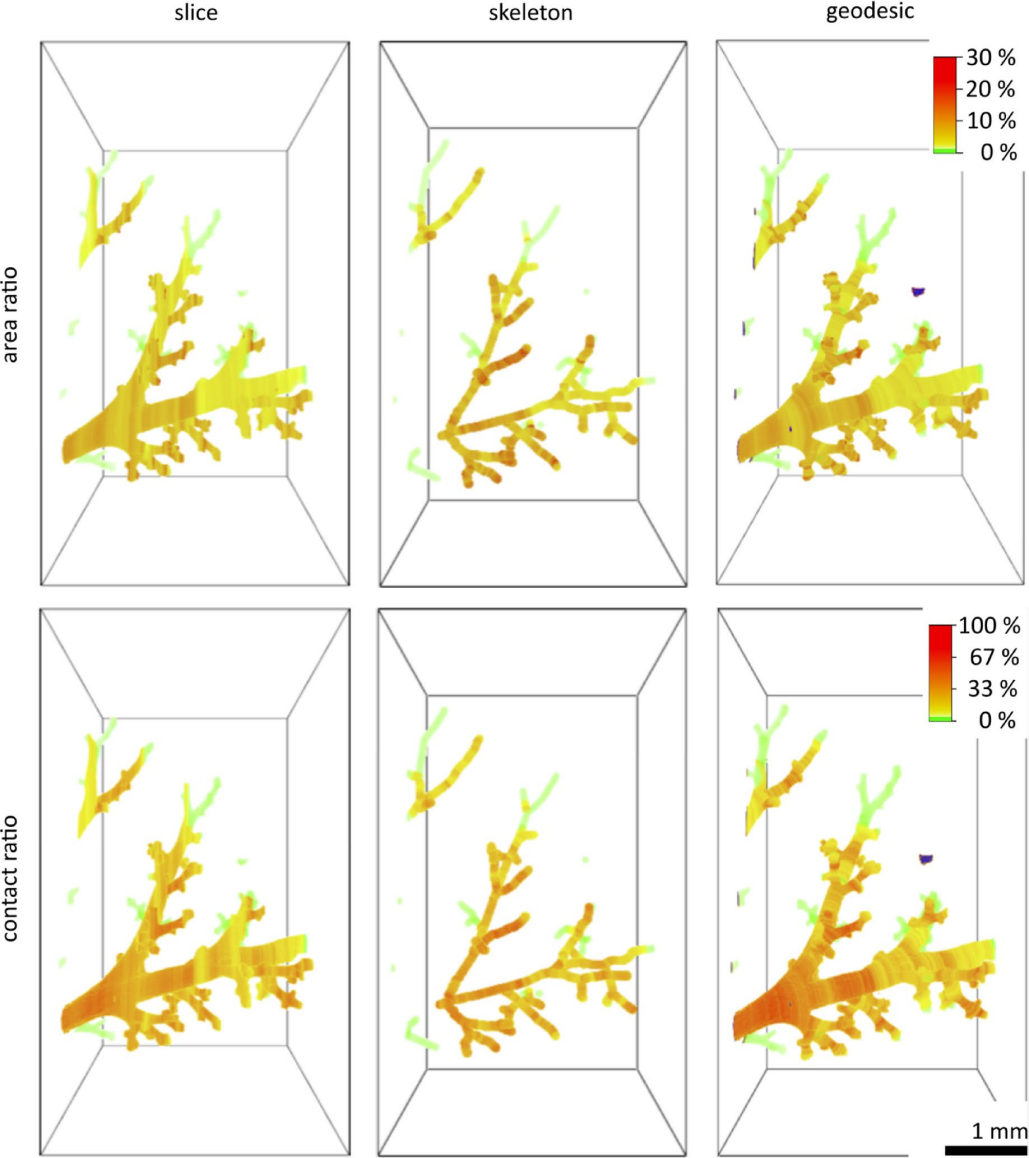
Localised microanalysis revealed spatial patterns of airway mucus obstruction in βENaC-tg mice. Representative images of microanalysis, which was performed on SRCT datasets acquired from selected sections of the left lung of an adult mouse from the βENaC-tg subset, providing high-resolution insights into the spatial distribution of mucus accumulation. An extensive post-processing pipeline was used to segment airways and mucus, allowing the calculation of two key parameters for each airway cross-section: the mucus area ratio and the mucus contact ratio. These parameters were calculated using three different mathematical methods (slice, skeleton, and geodesic), as detailed in the Methods section. The three-dimensional rendered images of the representative adult βENaC-tg mouse from the study subgroup visually represent the magnitude of these ratios along the airway tree, with a colour gradient ranging from green (lowest values) to red (highest values). In the geodesic method, unconnected components are highlighted in blue and were excluded from the analysis. Note the different ranges for the area (0–30%) and the contact ratio (0–100%). Scale bar: 1 mm. βENaC-tg = βENaC-transgenic. SRCT = synchrotron radiation-based computed tomography.

Localised microanalysis was performed, using additional SRCT imaging of selected areas of the murine left lung for the βENaC-tg study subset (5 juvenile, 5 adult), as described in Supplementary Table 1. Three computational methods (slice, skeleton, and geodesic) were used to analyse airway cross-sections. The slice method was fast, memory efficient, and yielded good results for airway branches parallel to the z-axis, however, it showed smearing for the other axis. The skeleton method was slow and memory intensive, but provided accurate cross-sections for main and secondary branches without smearing. However, it showed some topological inaccuracies due to the merging or omission of pixel data during rotation and skeletonisation. The geodesic method produced sharp-looking rendered images with moderate speed and memory requirements, but generated non-planar cross-sections and missed some unconnected airways for segmentation (Fig. 5 and Supplementary Fig. S3 online). Two key parameters were calculated for each airway cross-section, the mucus area ratio, as an indicator of the degree of luminal mucus obstruction, and the mucus contact ratio, as an indicator of mucus adherence to the airway wall. These ratios were visualised in separate 3D rendered images for each method. Figure 5 provides the results for a representative adult βENaC-tg mouse, whereas a comparison of the results for all βENaC-tg mice examined by SRCT are shown in Supplementary Fig. S3 online, both indicating similar results for all three computational methods.

Overall, a strong positive correlation was observed between the mean area ratio and the mean contact ratio in all three methods (*r* ≥ 0.80, Supplementary Fig. S4 online). Mean area ratios ranged from 0.7% to 15.5%, while mean contact ratios ranged from 6.4% to 58.1%, suggesting a wide spectrum of mucus obstruction severity. The linear relationship between these parameters, illustrated by the best-fit derived from simple linear regression (Y = 3,204*X + 7,243, R^2^ = 0.89, *P* < 0.0001), indicates a systematic and proportional increase in mucus airway wall adherence with increasing luminal obstruction (Supplementary Fig. S4 online).

### Comparative µCT-guided SEM facilitates nanoscale visualisation of mucus plug ultrastructure

**Fig. 6:**
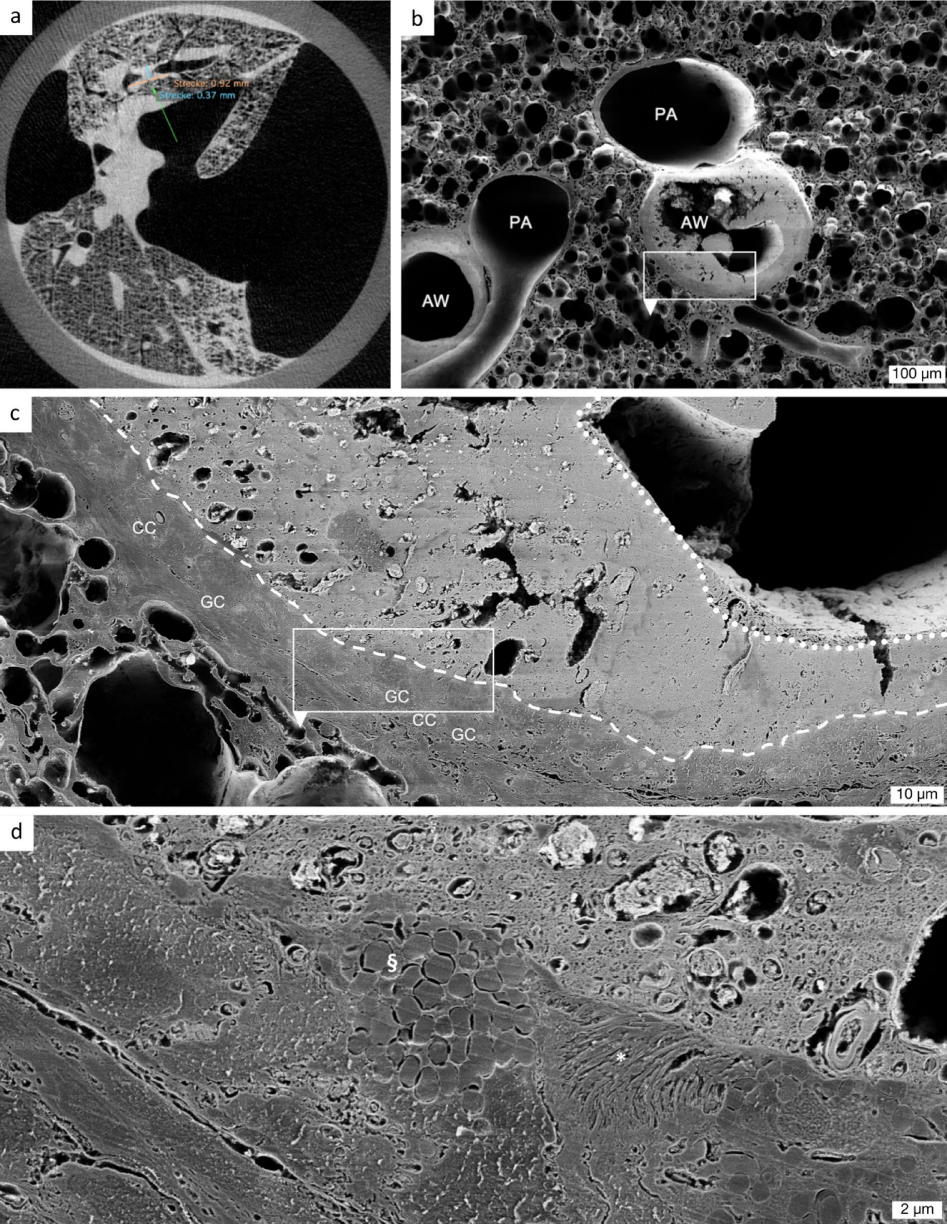
µCT-guided SEM reveals nanoscale ultrastructural details of airway mucus plugs. (a) Whole-lung µCT scan of a juvenile βENaC-tg mouse, used to localise a mucus plug within an airway as a region of interest (ROI) for downstream SEM. Following ROI identification, an optimal cutting plane (green line) was selected for subsequent SEM processing. (b) Low magnification SEM image of the ROI identified in (a), showing pulmonary arteries (PA) and airways (AW), one of which is occluded by a dense mucus plug. This view illustrates the overall lung architecture and the spatial relationship between airways and surrounding parenchyma. (c) High magnification SEM image of the mucus plug from the region highlighted in (b). (d) Nanoscale image of the region shown in (c), revealing a goblet cell with clustered mucin granules (§) and buried kinocilia (*) beneath a dense mucus layer. The mucus plug displays a well-defined border and a compact structure, and its finely preserved filamentous network, illustrates the cohesive nature of mucus plugs in βENaC-tg mice. SEM parameters: Working distance 5.4–5.5 mm, SE2 detector, and accelerating voltage 2 kV. Dashed line delineates border between epithelium and mucus. Stippled line delineates a superficial mucus compartment of different texture. Scale bars: 100 μm (b), 10 μm (c), and 2 μm (d). µCT = micro-computed tomography. SEM = scanning electron microscopy. βENaC-tg = βENaC-transgenic. CC = club cells. GC = goblet cells.

µCT-imaging of whole lungs was used to identify regions of interest for subsequent SEM, which was performed for the βENaC-tg study subset (3 juvenile), as described in Supplementary Table 2. This targeted approach enables precise correlation between radiological and ultrastructural findings. As shown in Fig. 6a, airway mucus plugs were localised in the µCT data sets, and ideal cutting planes (green line) were defined for downstream SEM processing. Low magnification SEM illustrates the overall lung architecture and the spatial relationship between airways and surrounding parenchyma (Fig. 6b). High magnification SEM revealed a stratified mucus plug architecture of complex, heterogeneous ultrastructure (Fig. 6c). The mucus matrix forms a highly compacted, cohesive, adherent basal layer that covers the airway epithelium directly (outlined by a dashed line, Fig. 6c). Numerous circular and irregularly shaped particles are entrapped within the matrix, consistent with impaired clearance and ongoing obstruction. Above the basal layer, a distinct superficial compartment displays a rough, filamentous texture, suggesting a different composition compared to the deeper core (outlined by a stippled line, Fig. 6c). At higher magnification, the interface between epithelium and mucus plug is sharply delineated, with buried kinocilia and a goblet cell visible beneath a dense mucus layer (Fig. 6d). The described ultrastructural organisation represents a characteristic example from our SEM dataset. This integrated imaging approach demonstrates that comparative µCT-targeted SEM facilitates nanoscale visualisation of the mucus plug ultrastructure. The combination of whole organ imaging with localised high-resolution SEM provides critical insights into the structural basis of airway obstruction in muco-obstructive pulmonary diseases.

## Discussion

This study employed a multimodal, high-resolution imaging approach to investigate the spatiotemporal dynamics of airway mucus plugging in βENaC-tg mice, a well-established model of muco-obstructive lung disease. By combining whole organ µCT, localised SRCT, and SEM, this study provides an integrated, multiscale analysis of airway mucus plugs across different developmental stages. Our pipeline bridges spatial scales from macro- to nanoscale, yielding new insights into the heterogeneous nature of mucus obstruction in this disease model. These findings build upon and advance previous observations by providing a quantitative, spatially resolved, and systematic assessment of mucus accumulation, thereby offering an important foundation for future therapeutic studies targeting mucus plugs in muco-obstructive lung diseases.

Consistent with previous research, βENaC-tg mice display a characteristic age-dependent progression of mucus obstruction^11,18–20^. Mucus plugging in this model has been described to originate predominantly in the trachea during the early neonatal period, contributing to significant neonatal mortality^18,19^. While the trachea was not included in the imaging analysis, the present study provides novel insights into the spatial distribution of intrapulmonary airway obstruction, revealing distinct regional and developmental patterns. Using a semiquantitative scoring system based on µCT data and an extended murine airway tree nomenclature encompassing 11 airway generations, we demonstrate that juvenile βENaC-tg mice exhibit the most extensive mucus accumulation, with a distribution across both proximal and distal airways. In contrast, adult βENaC-tg mice show persistent mucus obstruction concentrated in the proximal airways, with decreasing involvement toward the periphery. The distinct temporal pattern of mucus accumulation observed in this study mirrors the pathological transition described in previous histopathological and molecular studies. Specifically, the prominent mucus obstruction in juvenile mice aligns with the reported peak of IL-13–mediated type 2 inflammation and goblet cell metaplasia at this stage^18–20^. This transient inflammatory response is thought to prime the lungs for chronic airway remodelling, neutrophilic inflammation, and emphysematous lung destruction as the mice mature^7,18,21^. Our data are consistent with these earlier findings, while adding anatomical precision regarding the distribution of mucus plugs throughout the bronchial tree. Interestingly, our whole organ µCT-based scoring did not reveal significantly elevated mean mucus scores in newborn βENaC-tg mice when compared to age-matched wild-type controls. This result contrasts with previous µCT findings of Zhu et al.^22^, who reported increased numbers of mucus adhesion lesions in neonatal βENaC-tg lungs. Several factors may contribute to this apparent discrepancy. First, the inclusion of the trachea in their analysis likely contributed to the detection of early-stage obstruction, particularly since tracheal mucus plugging is a recognised hallmark of early disease in this model^18,19^. Second, methodological differences, such as fixation of the lungs at positive pressure rather than at residual lung capacity, and the absence of spatial resolution for detected lesions, may also account for discrepancies in the reported findings. This is further supported by a VCT study by Wielpütz et al.^19^, which distinguished between tracheal and main stem bronchi and demonstrated that newborn βENaC-tg mice predominantly exhibit tracheal mucus obstruction. Together, these studies highlight the importance of carefully distinguishing airway regions when interpreting imaging findings in muco-obstructive lung disease models. Of note, a regional heterogeneity of airway changes, including mucus plugging, can also be observed within individuals with chronic obstructive pulmonary disease (COPD), primary ciliary dyskinesia (PCD), or CF, which is often attributed to different mechanical properties of airway branching patterns, size, and regional chronic inflammation^27–29^. It is conceivable, that the different patterns of mucus accumulation in juvenile and adult βENaC-tg mice may allow the study of the influence of mechanical properties of the airway on mucus distribution.

Beyond confirming regional heterogeneity of mucus plugging, our study advances the field by quantitatively linking the extent of luminal obstruction to mucus adherence along airway wall surfaces. Using localised SRCT microanalysis, we identified a strong positive correlation between the mucus area ratio (an indicator of luminal obstruction) and the mucus contact ratio (a measure of airway wall adherence) across multiple computational approaches. This relationship suggests that, as mucus accumulates within the airway lumen, it adheres more strongly to the epithelial surfaces. Conversely, mucus may initially adhere to the airway wall, forming a base for subsequent accumulation and compaction within the lumen. This observation is biologically plausible, given that the known pathophysiology of mucus hyper-concentration and reduced airway surface hydration leads to mucus compaction and stronger adhesive interactions with epithelial surfaces, which impair mucociliary clearance and worsen airway obstruction^1–3,6,7,11,23^. Further supporting this interpretation, our SEM analyses revealed that airway mucus plugs exhibit a stratified ultrastructure, composed of distinct layers. The basal layer appears as a cohesive, compact matrix adherent to the airway epithelium, while the superficial layer looks more filamentous and structurally heterogeneous. It is important to acknowledge that these ultrastructural findings are based on a limited dataset from three juvenile βENaC-tg mice, representing a technically challenging and resource-intensive proof-of-concept methodology that combines precise spatial targeting with high-resolution analysis. While only limited regions per mouse were investigated due to technical constraints, the presented ultrastructure represents a characteristic example that demonstrates the feasibility of our correlative imaging approach. The observed mucus ultrastructure mirrors descriptions of stratified airway mucus not only in an elastase-exposed mouse model for COPD, but also in human COPD and CF patients, suggesting that βENaC-tg mice recapitulate key ultrastructural features of mucus pathology observed in muco-obstructive lung diseases^23^. Notably, the entrapment of particulate material within the mucus matrix, as visualised by SEM, further underscores the contribution of mucus plugging to impaired airway clearance and chronic inflammation. Despite the limited scope of our current SEM dataset, these findings establish the technical feasibility of targeted ultrastructural analysis in this animal model and provide initial insights that complement our comprehensive µCT and SRCT analysis.

The strong correlation between mucus accumulation and airway wall adherence, demonstrated in our study, carries important therapeutic implications. Addressing both, mucus viscosity and its adhesion to the airway epithelium, may offer a more comprehensive and effective therapeutic approach. Previous work has highlighted disulfide cross-linking between mucin polymers as a key mechanism promoting mucus viscosity and adhesion in muco-obstructive diseases^1,9,10,23,30^. Our findings therefore provide support for the development of reducing agents capable of cleaving these disulfide bonds to improve mucus mobilisation. Promising compounds in this regard include novel thiol-saccharide mucolytics, which have demonstrated efficacy in reducing mucus viscoelasticity and adhesion in preclinical models^9,10^. Additionally, surfactant therapy provides a complementary approach by reducing mucus-epithelium interactions and weakening mucus adhesion, potentially promoting the detachment and clearance of obstructive plugs^23,31^. Taken together, these findings may suggest that combining systemic therapy with cystic fibrosis transmembrane conductance regulator (CFTR) modulators and mucolytics could offer additional therapeutic benefits compared to CFTR modulators alone, potentially by promoting the regional detachment and clearance of mucus plugs.

An additional strength of our analysis approach lies in its hierarchical integration of imaging modalities. Whole-organ µCT provides comprehensive, airway segment-specific mucus scoring across the entire lung, while localised SRCT enables high-resolution, quantitative analysis of mucus distribution at the airway cross-section level. Finally, SEM offers nanoscale visualisation of mucus plug architecture. This correlative, multiscale strategy advances beyond prior single-modality approaches by contextualising anatomical patterns of mucus obstruction within both regional and ultrastructural frameworks. While functional imaging studies, such as those by Stahr et al.^25^, have demonstrated heterogeneous airflow restriction in βENaC-tg mice due to mucus obstruction, our work adds critical structural context by directly visualising the anatomical substrate underlying these functional impairments. Future studies should integrate functional and structural imaging techniques to provide a more holistic understanding of how regional mucus accumulation contributes to impaired pulmonary mechanics and gas exchange.

Despite its strengths, some limitations of our study should be acknowledged. First, although vascular perfusion fixation effectively preserved mucus in situ for imaging, sample preparation steps, including staining and agarose embedding, may introduce artefacts, such as structural alterations or partial detachment of mucus from airway surfaces. While our imaging findings were consistent across modalities, further validation using alternative fixation or in vivo imaging approaches could help confirm the integrity of observed mucus structures. Second, SRCT analyses were confined to randomly selected regions of the left lung due to technical and logistical constraints, limiting the ability to precisely correlate high-resolution findings with specific airway generations identified by µCT. Future methodological developments, including larger field of view SRCT or advanced segmentation algorithms, could help bridge this gap. Another limitation relates to the static, cross-sectional nature of our analysis. Although our multimodal imaging strategy provided unprecedented anatomical detail, it did not capture the dynamic processes of mucus plug formation, progression, or clearance over time. Longitudinal in vivo imaging studies using non-invasive modalities could provide valuable insights into the kinetics of mucus plug development and resolution, particularly in response to therapeutic interventions. Additionally, combining imaging data with molecular and immunological analyses could elucidate how airway mucus properties interface with inflammatory pathways, epithelial injury, and remodelling processes that drive chronic lung disease progression. Importantly, while our extended airway tree nomenclature facilitated detailed analysis of the murine bronchial architecture, caution must be exercised when extrapolating these findings to human disease due to anatomical differences between mouse and human lungs^18,26^. The βENaC-tg mouse model exhibits distinct regional patterns of initial mucus plugging compared to human muco-obstructive diseases such as CF, where obstruction typically initiates in small airways rather than proximal regions. As previously noted by Mall et al.^18^, these differences may reflect species-specific airway anatomy: mice possess limited airway branching, with the narrowest cross-sectional area occurring at the tracheal level, whereas humans exhibit extensive branching that creates critical surface area restrictions at the terminal bronchioles. Additional physiological differences include distinct temporal disease trajectories between species. In βENaC-tg mice, mucus plugging exhibits peak severity during the juvenile developmental period, whereas CF patients demonstrate progressive, cumulative deterioration of airway obstruction with advancing age. These species-specific characteristics limit the direct translation of anatomical mucus distribution patterns from mouse to human disease. Nevertheless, βENaC-tg mice remain a highly relevant preclinical model for studying muco-obstructive lung diseases and evaluating candidate therapeutics, as the fundamental pathophysiological mechanisms underlying airway surface liquid depletion and mucus concentration are highly conserved between the model and human disease.

Looking ahead, future research directions should focus on several key areas. First, refining in vivo imaging methodologies to enable serial assessments of mucus plug dynamics over time will be essential for developing robust preclinical models of therapeutic efficacy. Advances in µCT resolution, motion correction algorithms, and image registration could support this. Second, therapeutic studies incorporating targeted delivery of reducing agents or surfactants to regions of predominant mucus accumulation could help evaluate whether region-specific interventions offer additional clinical benefit to systemic therapies. Third, expanded ultrastructural SEM analysis across different airway generations and developmental stages represents an important avenue for future research that would enhance our understanding of mucus plug architecture and its relationship to disease progression. Finally, integrating advanced imaging approaches with emerging single-cell or spatial transcriptomic techniques could reveal the molecular and cellular contexts in which mucus plugs form, providing deeper mechanistic insights into disease pathogenesis.

In summary, our multimodal, high-resolution imaging framework provides a comprehensive and spatially resolved analysis of airway mucus obstruction in a model of muco-obstructive lung disease. By elucidating age- and region-specific patterns of mucus plugging, quantifying the relationship between luminal obstruction and airway wall adherence, and characterising plug ultrastructure, this study establishes a new methodological benchmark for investigating airway mucus characteristics. These findings emphasise the potential value of therapies targeting mucus adhesion and highlight the importance of considering anatomical heterogeneity in the pathogenesis and treatment of muco-obstructive lung diseases. Our correlative imaging platform lays the groundwork for future studies that bridge structural and functional insights, with the goal of informing novel interventions in muco-obstructive lung diseases.

## Methods

### Ethics declarations

All animal experiments were approved by the local competent authority Regierungspräsidium Karlsruhe and were conducted in compliance with the European Directive 2010/63/EU, the German Animal Welfare Act, the German Laboratory Animal Welfare Ordinance, and the ARRIVE guidelines.

### Experimental animals

βENaC-tg mice (also known as Scnn1b-tg) were used as a well-established animal model for muco-obstructive lung disease and were generated as previously described^11,12,18^. Female and male hemizygous βENaC-tg (*n* = 24) and wild-type (wt) littermate mice (*n* = 28) on the C57BL/6 background were used as experimental animals and negative controls, respectively. Genotyping was performed by polymerase chain reaction of genomic DNA, as previously described^11,18^. Mice were bred and maintained in the animal facility of Heidelberg University (Interfakultäre Biomedizinische Forschungseinrichtung, IBF) and housed under pathogen-free conditions, with access to food and water ad libitum. Mice were euthanised by an intraperitoneally administered overdose of ketamine and xylazine. Subjects were grouped by age, with newborn being 0 to 1 days, juvenile 13 to 16 days, and adult 49 to 59 days. Further characteristics of the study group are presented in Table 1.

### Lung fixation and staining

Immediately after euthanasia, mouse lungs were fixed using a vascular perfusion fixation technique, which involves modifications to a previously described protocol^22^ developed from the technique introduced by Vasilescu et al.^32^. In this technique, modifications were made to preserve the orthotopic localisation of mucus in the murine airways. The murine trachea was first exposed by tracheostomy in the supine position. The trachea was ligated with a surgical silk tie, without prior application of positive pressure to the airways via intratracheal cannulation. Tracheal ligation was performed to prevent lung deflation and to maintain lung inflation at residual lung capacity. The mouse chest was then opened by sternotomy. In the exposed mouse heart, a venous catheter was placed into the right ventricle, and the left atrium was opened by incision to allow drainage of blood and perfusion solutions. Two perfusion solutions were administered sequentially through the venous catheter into the pulmonary artery at constant physiological pressure. First, a pre-flush solution containing 5% dextran 70, 0.02% lidocaine, and 5 IU/mL heparin in Ringer’s solution was flushed for 15 min until all blood was drained from the murine pulmonary vasculature. Perfusion with a fixative solution containing 3.7% formaldehyde, 25% polyethylene glycol 400, and 10% ethanol in double distilled water was then continued for approximately 25 min, before the lung was removed and immediately immersed in fixative for at least 72 h. The fixed lung specimens were washed with distilled water, osmium-stained, lead aspartate-stained, and low melting agarose-embedded (see block staining protocol below).

### Micro-computed tomography (µCT)

Fixed, osmium-stained, and agarose-embedded samples were imaged using a SkyScan 1176 µCT scanner (Bruker). Image acquisition parameters were as follows: 9 μm isotropic voxel size, 50 kV source voltage, 500 µA source current, 1311 projection images, 5 frame averaging, 0.15° angular step over 180° rotation, 0.5 mm aluminium filter, 902 ms exposure time, and approximately 142 min total acquisition time. Image reconstruction was performed using NRecon software version 1.7.0.4 (Bruker; https://www.bruker.com/en/products-and-solutions/preclinical-imaging/micro-ct.html). The resulting 3D image data of the whole lung, with an isotropic voxel size of 9 μm, was used for subsequent visual mucus scoring.

### Visual mucus scoring

The extent and distribution of airway mucus obstruction throughout the mouse lung was assessed using a visual scoring system based on µCT image data sets. Multiplanar reconstructions were analysed using CTAn software version 1.10.0.0 (Bruker; https://www.bruker.com/en/products-and-solutions/preclinical-imaging/micro-ct.html). A radiologist with extensive experience in murine lung imaging, blinded to sample genotype, visually scored each individual airway segment on a scale of 0 to 2 (0 = no mucus, 1 = mucus obstructing up to 50% of the airway lumen, 2 = mucus obstructing more than 50% of the airway lumen). Airway segments were assigned to their respective airway generation and lung lobe using an extended murine airway tree nomenclature based on Thiesse et al.^26^ designed for monopodial airway branching. Our high-resolution µCT imaging method allowed us to extend this nomenclature by five additional airway generations, resulting in a total of 11 generations included in the visual mucus score (Fig. 3). In total, 9640 airway segments were individually scored, providing a comprehensive assessment of mucus obstruction patterns throughout the bronchial tree.

### Synchrotron radiation-based computed tomography (SRCT)

A subgroup of selected, fixed, osmium-stained, and agarose-embedded βENaC-tg lung samples underwent additional ultra-high-resolution SRCT imaging at the TOMCAT beamline of the Swiss Light Source (Paul Scherrer Institute)^33^. A section of each mouse left lung was imaged using a pco.Edge 5.5 detector (pco) in combination with a high-resolution microscope (Optique Peter). SRCT acquisition parameters were as follows: 3.25 μm voxel size, 18 keV beam energy, 400 mA ring current, 1501 projection images, 2.0x magnification, 0.120° angular step over 180.0° rotation, LuAG: Ce 20 μm scintillator, 50.0 ms exposure time, and approximately 4 min total acquisition time. Projection images were zero padded with a padding length of 0.5^34^. Image reconstruction was performed using the Fourier transform based Gridrec algorithm with Parzen filter^35^. The resulting 3D SRCT datasets of 5 juvenile and 5 adult βENaC-tg lung samples, with an isotropic voxel size of 3.25 μm, were used for post-processing, segmentation, and localised microanalysis. The characteristics of this subgroup are presented in Supplementary Table 1.

### Microanalysis of airway mucus

We developed a comprehensive post-processing pipeline using ToolIP software version 2019 (Fraunhofer ITWM; https://www.itwm.fraunhofer.de/en/departments/bv/products-and-services/toolip.html) and MAVI software version 1.5 (Fraunhofer ITWM; https://www.itwm.fraunhofer.de/en/departments/bv/products-and-services/mavi.html) to analyse 3D SRCT datasets from the βENaC-tg study subgroup.

Airways were segmented using a marked watershed and Tree Farm algorithm-based method, while airway mucus was segmented using binarisation and morphological operations (Supplementary Fig. S3 online). Airway mucus obstruction was quantified using two parameters: mucus area ratio and mucus contact ratio. The mucus area ratio represents the proportion of the airway lumen obstructed by mucus, whereas the mucus contact ratio represents the proportion of the airway wall covered by mucus. These ratios were calculated for each airway cross-section and mapped to create 3D visualisations (Fig. 5 and Supplementary Fig. S3 online).

We used three methods to calculate airway cross-sections. The slice method used original slices from airway segmentations, which were generally orthogonal to the main airway branch. The skeleton method generated airway skeletons using a thinning algorithm^36,37^, with cross-sections calculated orthogonal to the skeleton^38^. The geodesic method used the geodesic distance to the first slice containing the main airway branch. Cross-sections are then obtained as level sets of the geodesic distance transform. This method resulted in a 3D object rather than single, axis-aligned slices, effectively converting the mucus area ratio to a volume ratio. The calculation of the mucus contact ratio was adjusted accordingly. For simplicity, we continue to refer to these parameters as mucus area and contact ratio throughout the manuscript, despite their 3D nature in the geodesic method.

### Scanning electron microscopy (SEM)

Ultrastructural images of airway mucus plugs were obtained using SEM on selected juvenile βENaC-tg murine lung samples (*n* = 3). Following vascular perfusion fixation and block staining, specimens were embedded in agarose. Whole lung µCT scans were performed to identify airway mucus plugs as regions of interest, and to define optimal cutting planes for subsequent SEM analysis.

The lung samples were cut with a scalpel, after which 2.1 M sucrose was added, and the samples were frozen. A cryo-ultramicrotome was then used to obtain a smooth surface along the cut. The samples were then thawed and dehydrated in a series of ascending ethanol dilutions (70%, 95%, and 100%). After transferring the samples to acetone, the tissue blocks were placed in an automated critical point drier (Leica EM CPD300, Leica Vienna), which was set to perform 24 exchange cycles of carbon dioxide at medium speed with 20% stirring. All additional fill, heating, and venting steps were performed at a slow speed. After drying, the samples were mounted on EM-stubs using conductive silver.

Imaging was conducted using a Zeiss 1530 scanning electron microscope equipped with a Gemini column and ATLAS scan generator (Carl Zeiss, Oberkochen). The operating parameters included an accelerating voltage (EHT) of 1.50–2.92 kV and a working distance (WD) of 3.1–6.8 mm. Images were acquired using either the secondary electron detector or the InLens detector to provide ultrastructural details of airway mucus plugs (Fig. 6). The characteristics of this subgroup are presented in Supplementary Table 2.

### Block staining protocol

We used the reduced osmium tetroxide (OsO_4_)-thiocarbohydrazide-OsO_4_ (rOTO) method in conjunction with J. Walton’s lead aspartate^39,40^. Tissue blocks were incubated in 1.5% potassium ferrocyanide and 0.5% OsO_4_ in distilled water for 1 h. After washing three times for 20 min in distilled water, the samples were incubated in 1% freshly filtered thiocarbohydrazide for 40 min, followed by three rinses, 20 min each, with distilled water. Subsequently, the samples were incubated in 2% OsO_4_ in distilled water for 60 min at room temperature, and then washed with distilled water overnight at 4˚C. Finally, the samples were washed in 0.1 M cacodylic acid buffer, pH 5.5, for 20 min at room temperature, incubated with L-aspartate for 30 min at 60˚C. In detail: 0.998 g L-aspartate was dissolved in 250 mL doubly distilled water. The pH was adjusted with 1 N KOH to 3.8. Lead nitrate (0.66 g) was dissolved into 100 mL of L-aspartate solution and adjusted to pH 5.5 with 1 N KOH. Samples were washed twice in 0.1 M cacodylic acid buffer at pH 5.5, followed by two washes in 0.1 M cacodylic buffer at pH 7.4. The samples were washed three times for 20 min in doubly distilled water at room temperature.

### Statistical analyses and software

Statistical analyses were performed using GraphPad Prism software version 10.2.3 (GraphPad Software Inc.; https://www.graphpad.com). Mann-Whitney U tests were used to assess group differences between βENaC-tg and wild-type mice. Bonferroni-Dunn method was used to adjust significance values for multiple testing. For comparisons between the three age groups within the same genotype, the Kruskal-Wallis test was used, followed by Dunn’s post-hoc test for multiple comparisons. Pearson correlation coefficients were calculated, and simple linear regression was performed to assess relationships between variables. Statistical significance was defined as *P* < 0.05.

Data visualisation, including 3D rendering, was carried out using VGStudio Max software version 3.4.3 (Volume Graphics GmbH; https://volumegraphics.hexagon.com). Cross-section movies were generated using CTAn software version 1.20.8.0 (Bruker; https://www.bruker.com/en/products-and-solutions/preclinical-imaging/micro-ct.html). Video editing was performed using iMovie software version 10.4.2 (Apple Inc.; https://apple.com/imovie). Figures were assembled using Inkscape software version 1.2.2 (Inkscape Project; https://inkscape.org).

## Supplementary Information

Below is the link to the electronic supplementary material.

Supplementary Material 1

Supplementary Material 2

## Data Availability

The generated and analysed datasets are available from the corresponding author on reasonable request, due to their large size. Please refer to the online supplement to this manuscript for further information. Supplementary Video 1 is available online at the following link: https://osf.io/8czgh/files/xwafu.
