# Supplementary material for: High-resolution multimodal imaging reveals spatial and temporal heterogeneity of airway mucus plugging in mice with muco-obstructive lung disease
Source: Sci Rep. 2025 Nov 24;15:41760. doi: 10.1038/s41598-025-22537-7 (PMC12647124; doi:10.1038/s41598-025-22537-7)
Supplement: Supplementary file 1 — Supplementary Material 1 [file 41598_2025_22537_MOESM1_ESM.pdf]

High-resolution multimodal imaging reveals spatial and temporal heterogeneity of airway mucus plugging in mice with muco-obstructive lung disease

Claudia V. Benke*[1,2#] Julia Duerr[3,4,5#], Annika Engel[6,7], Christian Dullin[1,2,8,9], Wolfram Stiller[1,2], Heinz Horstmann[10], Claudia Redenbach[11], Maximilian Ackermann[12,13,14], Hans-Ulrich Kauczor[1,2], Thomas Kuner[2,10], Mark O. Wielpütz[1,2,15], Marcus A. Mall[3,4,5+], Willi L. Wagner[1,2,15+]

1. Department of Diagnostic and Interventional Radiology (DIR), Heidelberg University Hospital, Heidelberg, Germany
2. Translational Lung Research Center (TLRC), German Center for Lung Research (DZL), University Heidelberg, Heidelberg, Germany
3. Department of Pediatric Respiratory Medicine, Immunology and Critical Care Medicine, Charité - Universitätsmedizin Berlin, Corporate Member of Freie Universität Berlin and Humboldt-Universität, Berlin, Germany
4. German Center for Lung Research (DZL), Associated Partner Site Berlin, Berlin, Germany
5. German Center for Child and Adolescent Health (DZKJ), Partner Site Berlin, Berlin, Germany
6. Department of Mathematics, Technical University of Kaiserslautern (now part of RPTU University Kaiserslautern-Landau), Kaiserslautern, Germany
7. Hamburg University, Hamburg, Germany
8. Department of Diagnostic and Interventional Radiology, University Medical Center Goettingen, Goettingen, Germany
9. Translational Molecular Imaging, Max-Plank-Institute for Multidisciplinary Sciences, Goettingen, Germany

10. Department of Functional Neuroanatomy, Institute of Anatomy and Cell Biology, Heidelberg University, Heidelberg, Germany
11. Department of Mathematics, RPTU University Kaiserslautern-Landau, Kaiserslautern, Germany
12. Institute of Pathology, University Clinics of RWTH University, Aachen, Germany
13. Institute of Pathology and Molecular Pathology, Helios University Clinic Wuppertal, University of Witten/Herdecke, Witten, Germany
14. Institute of Anatomy, University Medical Center of the Johannes Gutenberg-University, Mainz, Germany
15. Diagnostic Radiology and Neuroradiology, University Medicine Greifswald, Greifswald, Germany

#equal contribution as first authors + equal contribution as senior authors

*Correspondence: Claudia.Benke@med.uni-heidelberg.de

SUPPLEMENTARY INFORMATION

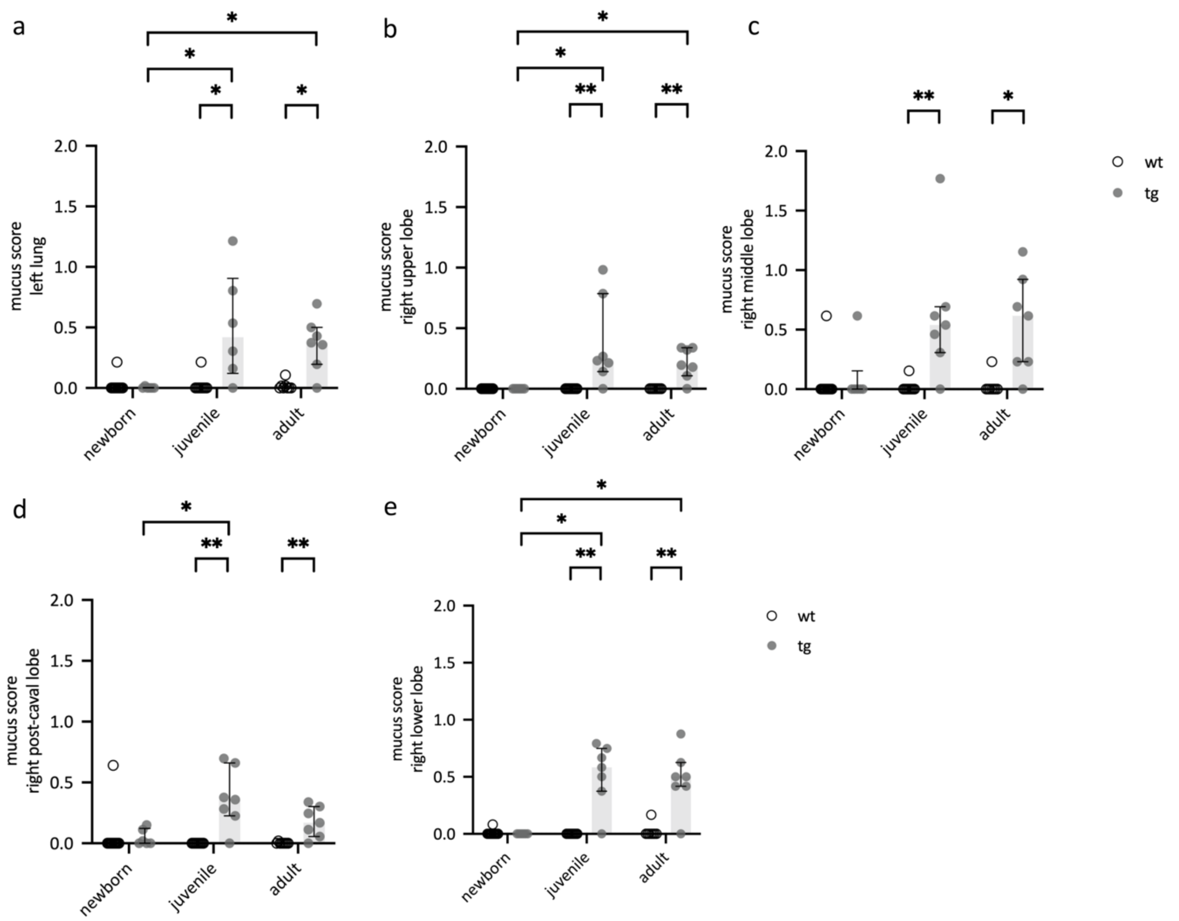

Supplementary Fig. S1. Age-dependent and lobe-specific airway mucus obstruction in βENaC-tg mice. Airway mucus obstruction across all lung lobes in βENaC-tg mice compared to wild-type controls at newborn, juvenile, and adult stages illustrate the heterogeneous nature of mucus plugging. Mean mucus scores derived from visual mucus scoring of µCT data sets are shown for the left lung (a), right upper lobe (b), right middle lobe (c), right post-caval lobe (d), and right lower lobe (e). Data are presented as median with interquartile range. wt = wild-type. tg = βENaC-transgenic. µCT = micro-computed tomography. n = 6-11 mice/group. **P < 0.05. **P < 0.01.*

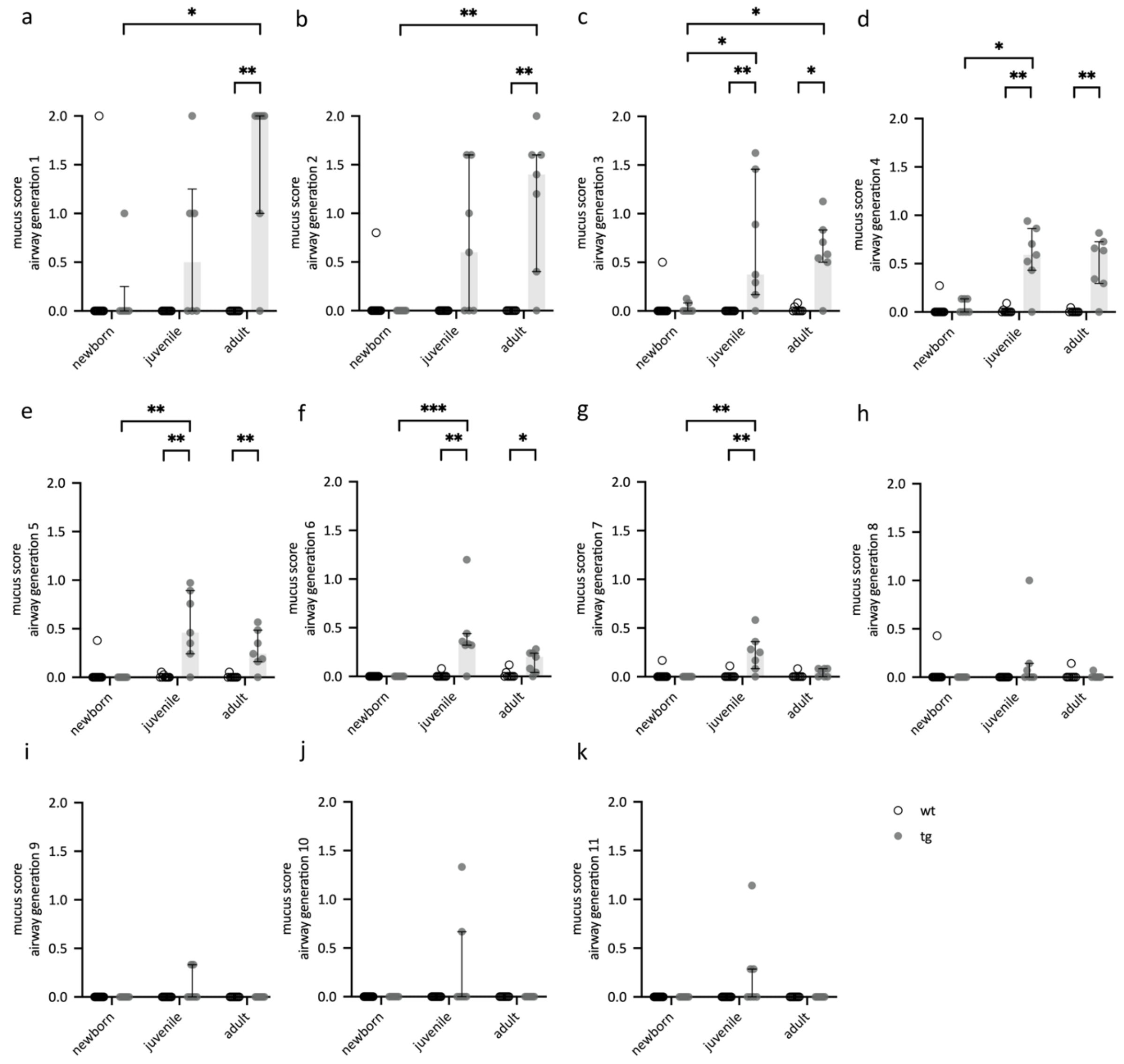

Supplementary Fig. S2. Airway generation-specific patterns of mucus obstruction at different ages in βENaC-tg mice. Mean mucus scores derived from visual mucus scoring of µCT data sets are shown across eleven airway generations (a-k) in βENaC-tg and wild-type mice at newborn, juvenile, and adult stages, revealing the complex spatio-temporal dynamics of mucus accumulation. Comparison between βENaC-tg and wild-type mice across different age groups highlights generation-specific patterns of muco-obstruction in the βENaC-tg mouse model. Data are presented as median with interquartile range. wt = wild-type. tg = βENaC-transgenic. µCT = micro-computed tomography. n = 6-11 mice/group. **P < 0.05. **P < 0.01. ***P < 0.001.*

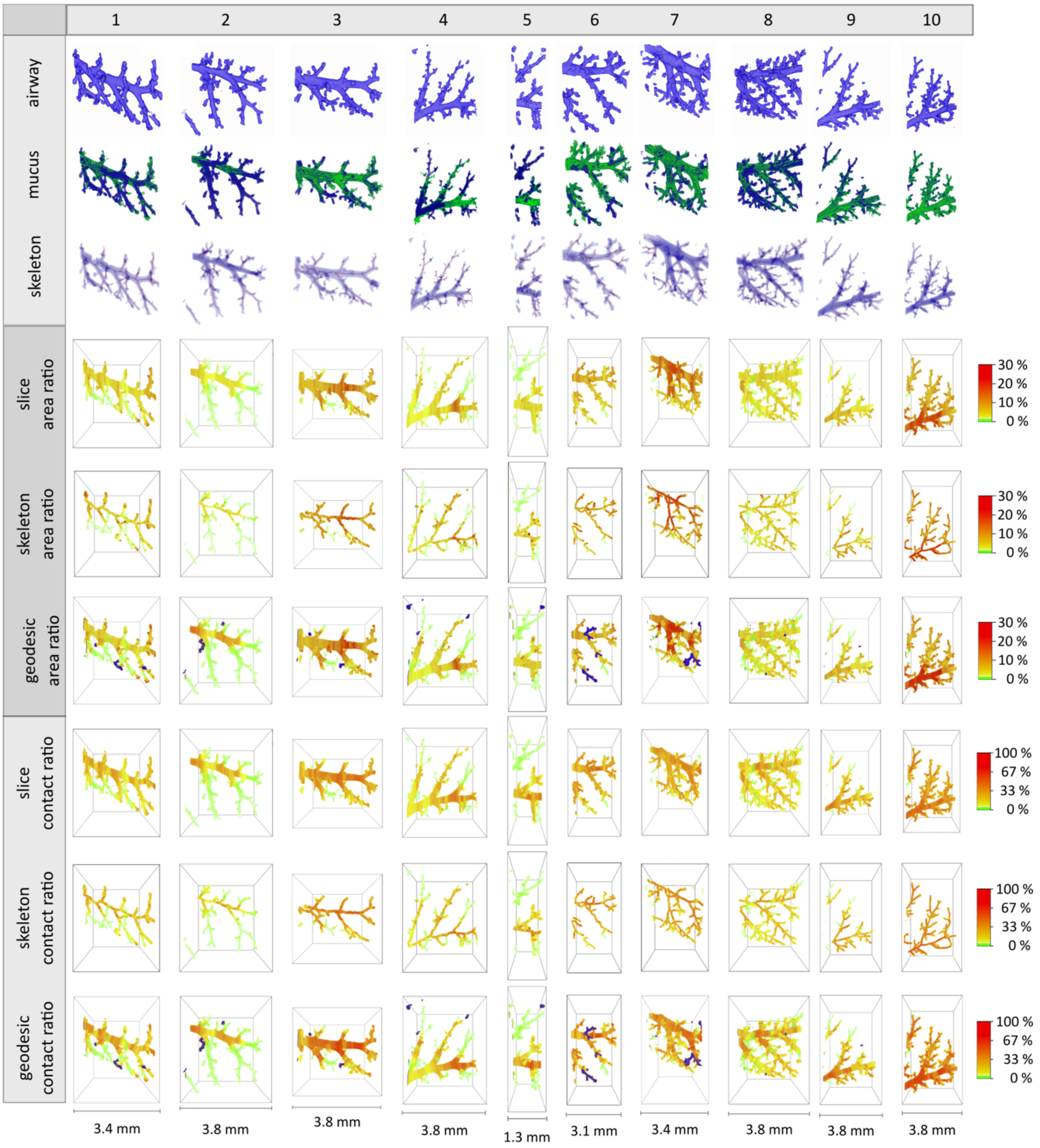

Supplementary Fig. S3. Overview of the localised microanalysis of airway mucus in the βENaC-tg study subset. Microanalysis of airway mucus obstruction using three-dimensional SRCT image datasets from the βENaC-tg study subset (Supplementary Table 1). For each individual mouse, the figure shows the airway segmentations (blue) in the "airway" row, combined airway (blue) and airway mucus (green) segmentations in the "mucus" row, generated airway skeletons (blue) in the "skeleton" row, area and contact ratios calculated using three methods (slice, skeleton, and geodesic), with colour-coded visualisations (area ratio: green 0% to red 30%; contact ratio: green 0% to red 100%) in the following rows respectively. The geodesic method highlights unconnected components in blue, which

were excluded from the analysis. All SRCT datasets were acquired from sections of the left lung. βENaC-tg = βENaC-transgenic. SRCT = synchrotron radiation-based computed tomography.

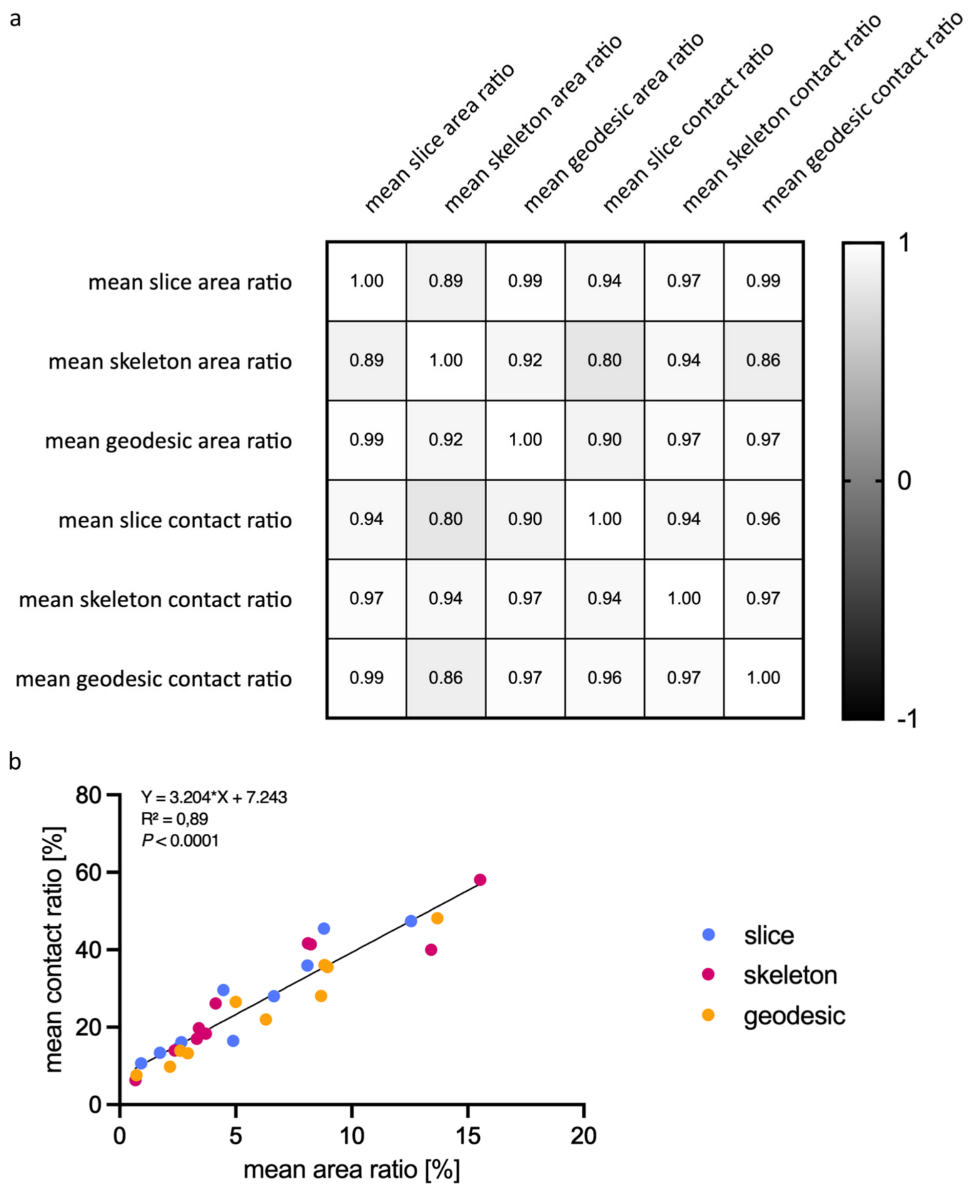

Supplementary Fig. S4. Mucus airway wall adherence positively correlates with the degree of luminal mucus obstruction. (a) A heat map illustrates the strong positive correlation between mean mucus area and contact ratios obtained from the analysis of SRCT image data in the βENaC-tg study subset (Supplementary Table 1), using the slice, skeleton, and geodesic methods. Pearson correlation coefficients ranged from -1 (black, high negative correlation) to 1 (white, high positive correlation). (b) A scatterplot further visualises this positive correlation, with data points from all three methods (blue: slice, pink: skeleton, and orange: geodesic) showing a linear trend, illustrated by the simple linear regression fit lined. Mean area ratios range from approximately 0-15%, while mean contact ratios ranged from 0-60%. The consistent distribution of data points across the measurement range suggests

a systematic relationship between mucus airway wall adherence and luminal obstruction, providing insight into the nature of mucus accumulation in this model of muco-obstructive lung disease. SRCT = synchrotron radiation-based computed tomography. βENaC-tg = βENaC-transgenic.

Supplementary Table 1. Characteristics of βENaC-tg study subset used for SRCT. Overview of the characteristics of the βENaC-tg lung samples selected from the total study group, whose SRCT image data were used for further post-processing, segmentation, and localised microanalysis, as described in the Methods section. Age and weight data are presented as mean ± standard deviation. βENaC-tg = βENaC-transgenic. SRCT = synchrotron radiation-based computed tomography.

	βENaC-tg	
age group	juvenile	adult
number of mice	5	5
age [days]	13.6 ± 0.5	54.8 ± 5.3
female/male/unknown	3/2/0	2/3/0
weight [g]	6.9 ± 0.6	23.2 ± 2.8

Supplementary Table 2. Characteristics of βENaC-tg study subset used for SEM. Overview of the characteristics of the βENaC-tg lung samples selected from the total study group, whose SEM image data were used for ultrastructural analysis, as described in the Methods section. Age and weight data are presented as mean ± standard deviation. βENaC-tg = βENaC-transgenic. SEM = scanning electron microscopy.

	βENaC-tg
age group	juvenile
number of mice	3
age [days]	14.7 ± 1.2
female/male/unknown	0/0/3
weight [g]	unknown
